# Monocyte Chemoattractant Protein-1 Deficiency Attenuates Oxidative Stress and Protects against Ovariectomy-Induced Chronic Inflammation in Mice

**DOI:** 10.1371/journal.pone.0072108

**Published:** 2013-08-19

**Authors:** Woon-Ki Kim, Eun-Kyung Choi, Ok-Joo Sul, Yeon-Kyung Park, Eun-Sook Kim, Rina Yu, Jae-Hee Suh, Hye-Seon Choi

**Affiliations:** 1 Department of Biological Sciences, University of Ulsan, Ulsan, Korea; 2 Department of Endocrinology, Ulsan University Hospital, Ulsan, Korea; 3 Department of Food Science and Nutrition, University of Ulsan, Ulsan, Korea; 4 Department of Pathology, Ulsan University Hospital, Ulsan, Korea; The Chinese University of Hong Kong, Hong Kong

## Abstract

**Background:**

Loss of ovarian function is highly associated with an elevated risk of metabolic disease. Monocyte chemoattractant protein-1 (MCP-1, C-C chemokine ligand 2) plays critical roles in the development of inflammation, but its role in ovariectomy (OVX)-induced metabolic disturbance has not been known.

**Methodology and Principal Findings:**

We investigated the role of MCP-1 in OVX-induced metabolic perturbation using MCP-1-knockout mice. OVX increased fat mass, serum levels of MCP-1, macrophage-colony stimulating factor (M-CSF), and reactive oxygen species (ROS), whereas MCP-1 deficiency attenuated these. OVX-induced increases of visceral fat resulted in elevated levels of highly inflammatory CD11c-expressing cells as well as other immune cells in adipose tissue, whereas a lack of MCP-1 significantly reduced all of these levels. MCP-1 deficiency attenuated activation of phospholipase Cγ2, transforming oncogene from Ak strain, and extracellular signal-regulated kinase as well as generation of ROS, which is required for up-regulating CD11c expression upon M-CSF stimulation in bone marrow-derived macrophages.

**Conclusions/Significance:**

Our data suggested that MCP-1 plays a key role in developing metabolic perturbation caused by a loss of ovarian functions through elevating CD11c expression via ROS generation.

## Introduction

The prevalence of metabolic syndrome increases with menopause. A loss of ovarian function is associated with increased fat, along with metabolic pathologies including insulin resistance (IR) and type 2 diabetes (T2D) [1]. The fact that increasing numbers of postmenopausal women expect longer life spans requires an understanding of the pathologies and molecular mechanisms of metabolic diseases associated with menopause. Ovariectomy (OVX) in rodent is considered to resemble human menopause. OVX results in increase of fat mass [2], [3] and chronic inflammation accompanied by IR [4], [5], [6], [7], implying a connection between metabolic complications observed in OVX and obesity. Chronic inflammation, which simultaneously occurs in obesity, contributes to IR and T2D [8]. Adipose tissue macrophage (ATM) which accumulates in adipose tissue (AT) with increasing body weight, is suggested as responsible for chronic inflammation, resulting in mediating IR [8]. This suggestion is supported by findings that increased ATM is associated with an aggravation of insulin sensitivity [9], whereas decreased ATM reduces IR caused by high fat diet (HFD)-induced obesity [10].

Tremendous heterogeneity is observed with ATM according to the local metabolic microenvironment. There are two populations of macrophages, the one of which expresses CD11c, an M1 macrophage marker, is suggested to be specifically recruited to AT upon HFD [11]. M1 ATM produces tumor necrosis factor-α, interleukin (IL)-6, and monocyte chemoattractant protein-1 (MCP-1), which induce IR, suggesting a connection between CD11c and IR. M2 ATMs have a different expression pattern with high levels of CD163, arginase-1, or IL-10, which are mainly associated with tissue repair [12].

MCP-1 (CCL2) plays critical roles in the development of inflammation and the recruitment of immune cells to the site of inflammation. In HFD-induced obesity, MCP-1 is highly expressed in AT, and circulating concentration is elevated, whereas administration of thiazolidinedione decreases MCP-1 levels [13], [14]. During menopausal transition, circulating level of MCP-1 has increased significantly, suggesting a role of MCP-1 as an indicator of hormonal change [15]. Increased serum MCP-1 level in humans correlates with markers of metabolic disorder including obesity, IR, and T2D [16]. Indeed MCP-1 inhibits insulin-induced glucose uptake [17]. In addition, high glucose levels induce monocytes to produce MCP-1 [18]. These results suggest a critical role of MCP-1 in inflammation associated with metabolic dysfunction. On the contrary, Park et al shows no correlation between circulating level of MCP-1 and obesity or IR [19].

In this study, we investigated the role of MCP-1 in OVX-induced metabolic perturbation, which is associated with CD11c-expressing ATM via up-regulation of ROS.

## Results

### MCP-1 Deficiency Decreases Visceral Adiposity and Metabolic Perturbation Induced by OVX

**Figure 1:**
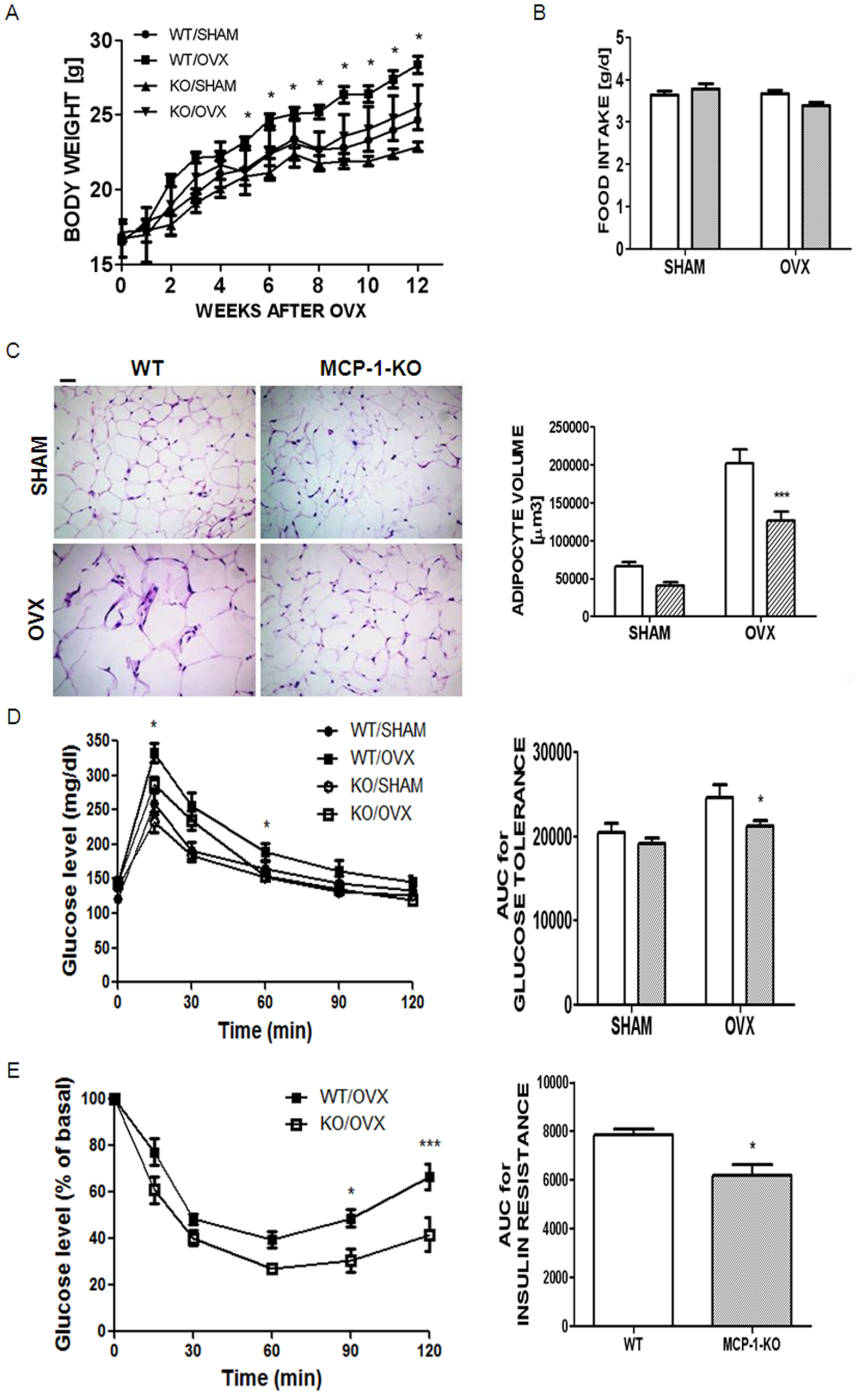
The absence of MCP-1 reduced fat mass and improved metabolic perturbation induced by OVX. WT mice (open bar) and MCP-1-KO (oblique-lined bar) mice were subjected to OVX or sham surgery, and then held for 12 weeks. Whole body weight change up to 12 weeks after surgery (A) and average daily food intake (B) were measured. *, *P*<0.05; WT OVX vs. MCP-1-KO OVX mice. Adipocyte volume was calculated from photograph of hematoxylin-eosin staining of visceral fat, assuming that an adipocyte is a sphere (magnification, ×200). Scale bar, 100 µm (C). Glucose clearance (D) and insulin sensitivity (E) were determined 12 weeks after sham or OVX, following an intraperitoneal injection of glucose (1 mg/kg) and insulin (0.75 munits/kg), respectively. AUC was measured for each group for D and E. *, *P*<0.05, ***, *P*<0.001; WT OVX vs. MCP-1-KO OVX. Data are expressed as mean ± SEM. Differences between groups were analyzed by two-way ANOVA, followed by Bonferroni post-tests (C, D) (adipocyte volume; *P*<0.001, AUC for glucose tolerance; *P*<0.01, effect of surgery. adipocyte volume; *P*<0.001, AUC for glucose tolerance; *P*<0.05, effect of MCP-1). *, *P*<0.05; ***, *P*<0.001 compared with WT OVX mice. Similar results were obtained in three independent experiments.

In previous studies, we demonstrated that OVX increases serum MCP-1 level [20]. To investigate whether MCP-1 contributes to OVX-induced metabolic dysfunction, we analyzed MCP-1-knockout (KO) mice, compared with wild type (WT) mice. The body weight increase of MCP-1-KO mice after sham surgery was slightly lower than WT mice at 18 weeks old (5.9±0.47 g vs. 3.9±0.44 g, respectively), but not significantly (**Figure 1A** **,** **Table 1**). In addition, no significant difference was found in subcutaneous fat or visceral fat between the two sham surgery groups, thus showing that MCP-1 did not affect fat mass in the sham surgery mice (**Table 1**). Next, we investigated whether the absence of MCP-1 affected OVX-induced metabolic disorder. Not only body weight, but also fat masses of visceral (2.10-folds) and subcutaneous AT were significantly reduced in MCP-1-KO mice compared to WT mice 12 weeks after OVX (**Table 1**). However, neither OVX nor MCP-1 induced a significant difference in daily food intake (**Figure 1B**), suggesting that an altered metabolic rate may be responsible for the increased fat mass and body weight induced by OVX. Histological examination of visceral fat revealed that OVX markedly increased adipocyte volume and the absence of MCP-1 significantly reduced it (1.61-folds), whereas no significant difference was obtained between sham surgery groups (**Figure 1C**). Twelve weeks after OVX, we observed a 1.9-fold increase in serum MCP-1 level compared with the sham surgery mice (**Table 1**). Serum levels of M-CSF and H_2_O_2_, which were elevated after OVX, were significantly attenuated in the absence of MCP-1 (**Table 1**). MCP-1 deficiency reduced elevated blood glucose level induced by OVX (**Table 1**). To confirm that the absence of MCP-1 improved glucose tolerance, which was ameliorated by OVX, we next determined glucose clearance following an intraperitoneal injection of glucose. In these experiments, the glucose tolerance in the sham surgery mice was not significantly affected by genotype. However, glucose levels decreased significantly after glucose injection in OVX MCP-1-KO mice, compared with OVX WT mice, indicating that the absence of MCP-1 improved glucose tolerance after OVX (**Figure 1D**). The absence of MCP-1 significantly decreased the area under curve (AUC) for glucose tolerance in OVX mice. Similarly, glucose was cleared more effectively in OVX MCP-1-KO mice than in OVX WT mice after an intraperitoneally-injected insulin as a measure of insulin sensitivity (**Figure 1E**). Taken together, these data showed that lack of MCP-1 improved insulin sensitivity in OVX mice.

**Table 1:** Physiological measurements of sham and OVX of WT and MCP-1-KO mice 12 weeks after operation.

Variable	WT		MCP-1-KO	
	SHAM	OVX	SHAM	OVX
Increased body weight [g]	5.891±0.4724	12.41±0.6270	3.928±0.4435	9.273±0.9983**
Subcutaneous fat [mg]	183.2±44.56	796.4±129.8	159.8±21.65	319.0±34.53**
Visceral fat [mg]	335.4±12.81	856.7±89.65	247.1±26.10	408.0±43.29***
Serum MCP-1[pg/ml]	28.80±3.625	54.71±8.922	ND	ND
Serum M-CSF [pg/ml]	40.95±1.388	52.29±1.703	36.68±1.347	40.81±2.933**
Serum H_2_O_2_ [nmol/ml]	48.63±1.105	59.72±1.432	46.98±2.580	51.74±0.6872***
Blood glucose [mg/dl]	110.2±2.619	130.1±4.713	114.7±5.413	112.1±4.810**
Blood insulin [ng/ml]	0.6767±0.01936	0.8313±0.02216	0.6050±0.01979	0.7471±0.03183*

Data are expressed as mean ± SEM. ND, non-detectible. Differences between groups were analyzed by two-way ANOVA, followed by Bonferroni post-tests (Increased body weight, subcutaneous fat, visceral fat, serum H_2_O_2_, and blood insulin; *P*<0.001, serum M-CSF; *P*<0.01, blood glucose; *P*<0.05, effect of surgery. Increased body weight, subcutaneous fat, and visceral fat; *P*<0.001, serum M-CSF and blood insulin; *P*<0.01, serum ROS; *P*<0.05, effect of MCP-1). WT OVX vs. MCP-1-KO OVX;

* *P*<0.05,

** *P*<0.01,

*** *P*<0.001.

### MCP-1 Deficiency Attenuates OVX-induced CD11c-expressing M1 Macrophages in AT

**Figure 2:**
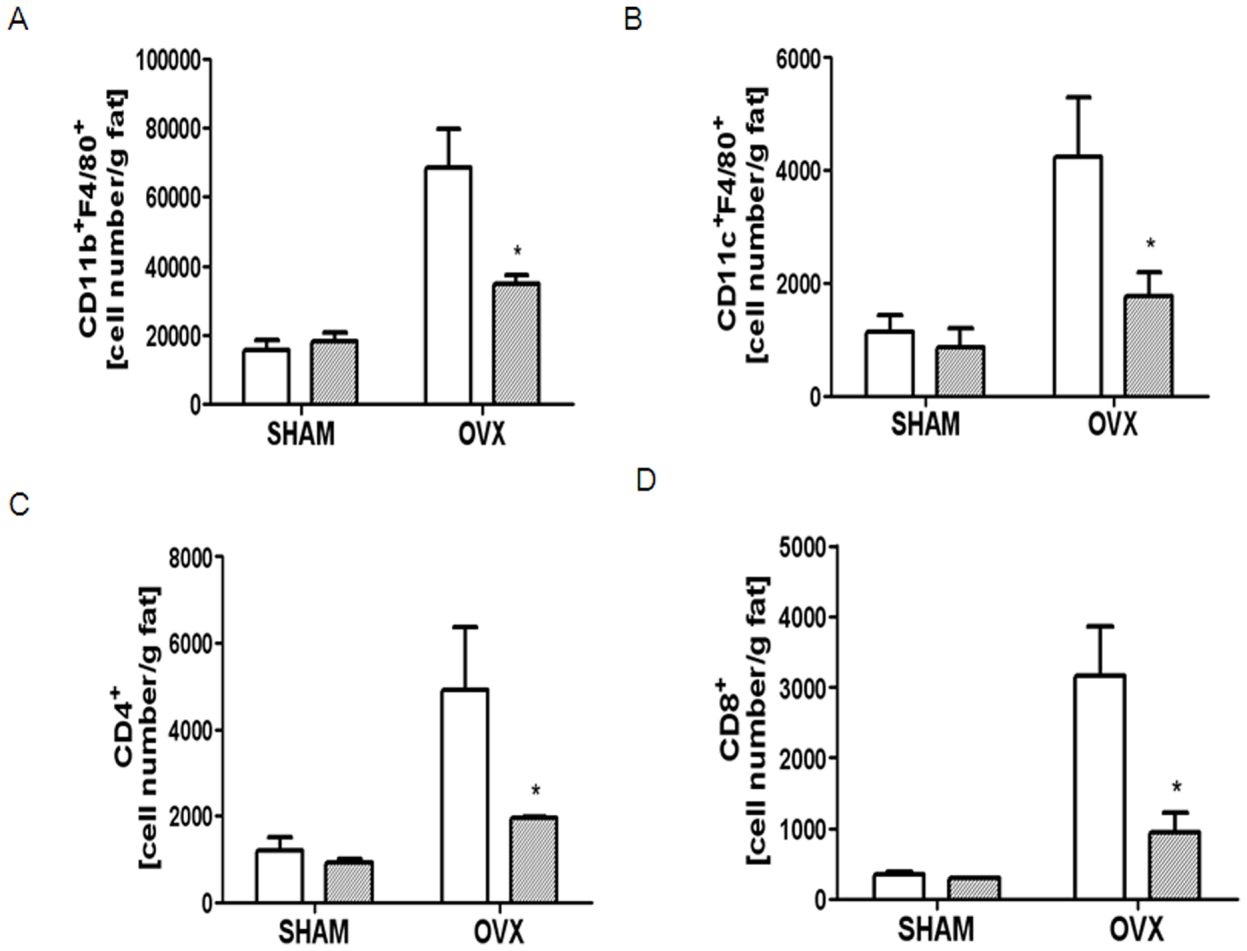
MCP-1-deficiency decreased OVX-induced immune cell infiltration in AT. SVCs from visceral fat were extracted from WT (open bar) and MCP-1-KO mice (oblique-lined bar) 12 weeks after sham or OVX surgery. SVCs were labeled with conjugated Abs to CD11bF4/80 (A), CD11cF4/80 (B), CD4 (C), and CD8 (D) and quantified by flow cytometry. Data are expressed as mean ± SEM. Differences between groups were analyzed by two-way ANOVA, followed by Bonferroni post-tests (CD11bF4/80, CD11cF4/80, CD4; *P*<0.01, CD8; *P*<0.05, effect of surgery. CD11cF4/80; *P*<0.05, effect of MCP-1). *, *P*<0.05; **, *P*<0.01; ***, *P*<0.001 compared with WT OVX mice. Similar results were obtained in three independent experiments.

The findings that ATM that accumulates during obesity results in IR [21] and that MCP-1mediates the recruitment of macrophages in tissues [9] prompted us to further investigate the association of the increased insulin sensitivity observed upon OVX in the absence of MCP-1 with decreased ATM. To determine whether MCP-1-deficient mice were protected from AT inflammation induced by OVX, we evaluated visceral fat pad stromal vascular cells (SVC) using flow cytometry. A profound increase in ATM number in OVX WT mice was found; however, while the visceral fat isolated from MCP-1-deficient mice accumulated strikingly fewer ATM, the level was not completely restored to that of the sham WT mice (**Figure 2A**). As shown in **Figure 2B–D**, OVX increased cell numbers of CD11cF4/80, CD4, and CD8 in WT mice, whereas the lack of MCP-1 significantly decreased all of these cells, showing that MCP-1 plays a critical role in elevating immune cells in AT upon OVX.

**Figure 3:**
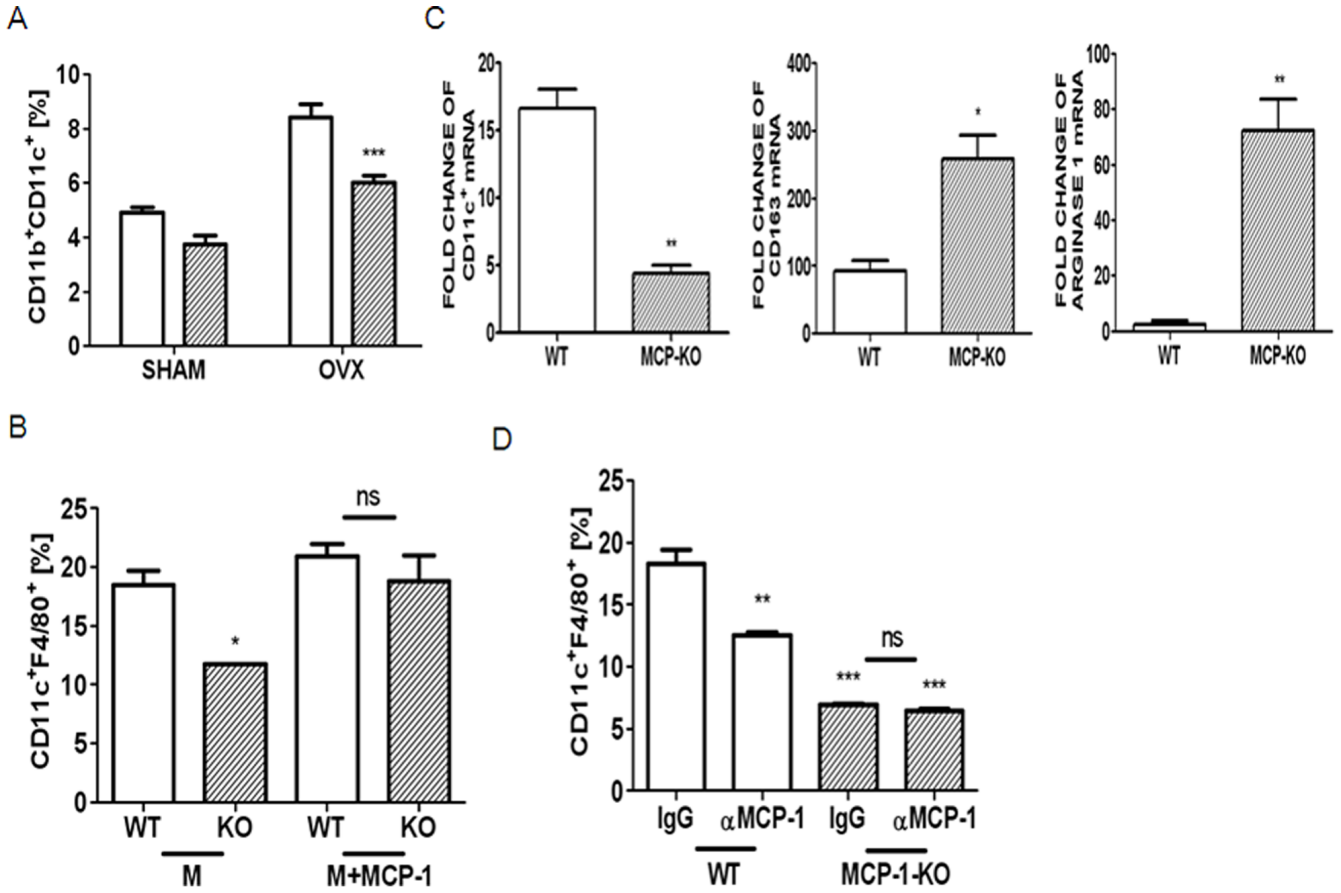
MCP-1-deficiency decreased CD11c upon M-CSF stimulation in BMM as well as an influx of CD11c. CD11b+CD11c+ monocytes in blood of WT (open bar) and MCP-1-KO mice (oblique-lined bar) 12 weeks after sham or OVX surgery were quantified (A). Data are expressed as mean ± SEM. Differences between groups were analyzed by two-way ANOVA, followed by Bonferroni post-tests. *P*<0.001, effect of surgery. *P*<0.001, effect of MCP-1. ***, *P*<0.001 compared with WT OVX mice. BMMs from WT and MCP-1-KO mice were stimulated using M-CSF (30 ng/ml) with or without MCP-1 (30 ng/ml) for 4 d and labeled with conjugated Abs to CD11c and F4/80 combination to quantify by flow cytometry (B). *, *P*<0.05 compared with M-CSF-treated WT cells. There was no significant difference in MCP-1-KO cells. BMMs were stimulated with M-CSF for 3 d, and total RNA was extracted and subjected to qPCR analysis (C). The expression level before M-CSF treatment was set to be 1. *, *P*<0.05; **, *P*<0.01 as compared with WT cells. BMMs from WT and MCP-1-KO mice were incubated in the presence of M-CSF with control IgG (3 µg/ml) or anti-MCP-1 Ab (3 µg/ml) for 4 d (D). **, *P*<0.01; ***, *P*<0.001 compared with IgG-treated WT cells. There was no significant difference in MCP-1-KO cells. Similar results were obtained in three independent experiments.

Since CD11c-expressing macrophages are responsible for chronic inflammation and insulin sensitivity [22], we focused our investigation on the precise mechanism of how MCP-1 modulates the increase in CD11c macrophages in AT. First, we determined whether the increase is due to an increased influx of CD11c-expressing monocytes in AT, an elevated differentiation and activation of bone marrow-derived precursors into CD11c-expressing M1 macrophages in residential tissues, or both. Using CD11b as a marker of mouse monocytes, we classified blood monocytes as CD11c+ or CD11c− monocytes. A significant increase in the percentage of CD11c monocytes in total leukocytes was observed in OVX mice compared with sham surgery mice (**Figure 3A**). MCP-1 deficiency reduced blood CD11c cells after OVX compared with WT mice, suggesting that MCP-1 is responsible for recruiting CD11c monocytes in AT. Next, to assess whether MCP-1 affects the phenotypes of macrophages during differentiation and activation in residential tissues, bone marrow-derived precursors were exposed to M-CSF. After 4 d exposure of M-CSF on BMM, about 18% of total cells expressed CD11c and F4/80, but the lack of MCP-1 significantly reduced this percentage (**Figure 3B**). To analyze the characteristics of M-CSF-stimulated cells, we compared transcript levels for specific markers for WT cells with those of MCP-1-KO cells. M-CSF stimulation induced a significantly reduced level of CD11c with increased CD163 and arginase 1 in MCP-1-KO cells, compared to WT cells (**Figure 3C**), demonstrating that the absence of MCP-1 reduced the ability of bone marrow-derived macrophage (BMM) to express M1-specific characteristics upon M-CSF stimulation. To assess whether the elevated expression of CD11c is due to MCP-1, we examined the effect of neutralization of MCP-1. Blockage of MCP-1 reduced the percentage of WT cells expressing CD11C upon M-CSF stimulation, whereas no further decrease was found in the absence of MCP-1 (**Figure 3D**). Next, complete restoration of CD11c was found in MCP-1-KO BMM upon stimulation of combination of M-CSF and MCP-1, whereas the combination did not significantly increase CD11c, compared with M-CSF alone in WT BMM (**Figure 3B**).

### MCP-1 Up-regulates CD11c Expression via Generation of Reactive Oxygen Species (ROS) and Fortification of the Activation of PLCγ2, Akt, and ERK upon M-CSF Stimulation

**Figure 4:**
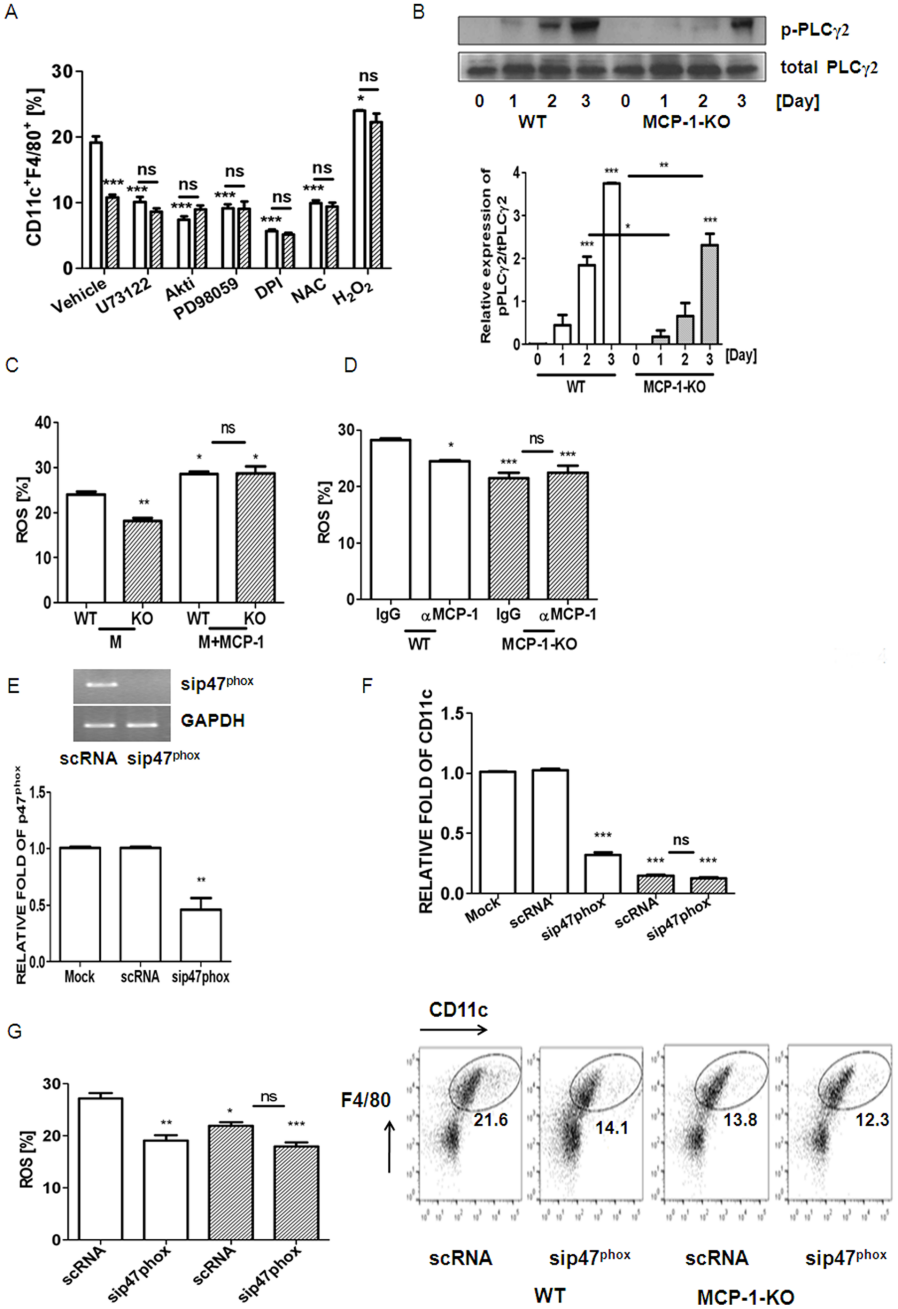
MCP-1-deficiency decreased CD11c-expressing cells via impairing the production of ROS and decreased activation of PLCγ2, Akt, and ERK upon M-CSF stimulation in BMM. BMMs from WT (open bar) and MCP-1-KO mice (oblique-lined bar) were incubated in the presence of M-CSF (30 ng/ml) with U73122 (10 µM), Akt inhibitor IV (0.3 µM), PD098059 (5 µM), DPI (50 nM), NAC (3 mM), or H_2_O_2_ (300 µM) for 4 d (A). *, *P*<0.05; ***, *P*<0.001 compared with vehicle-treated WT cells. There was no significant difference between WT and MCP-1-KO cells except upon vehicle treatment. Treatment with U73122, Akt inhibitor IV, PD98059, DPI, NAC, or H_2_O_2_ abolished the decrease in CD11cF4/80 observed in MCP-1-KO cells. BMMs were serum-starved for 8 h and stimulated with M-CSF for 1, 2, or 3 d (B). Phosphorylation of PLCγ2 was determined by Western blotting. Total protein level served as the loading control. Relative ratios of phosphorylated forms to total forms were plotted. **, *P*<0.01; ***, *P*<0.001 compared with WT cells. Intracellular levels of ROS upon stimulation in the presence of M-CSF (M) or/and MCP-1 with control IgG (3 µg/ml) or anti-MCP-1 Ab (3 µg/ml) for 2 d were determined in WT cells and MCP-1-KO cells using H2DCFDA (C, D). ROS levels were quantified by flow cytometry. *, *P*<0.05; **, *P*<0.01 compared with M-stimulated WT cells. No significant difference between WT and MCP-1-KO cells stimulated with M+MCP-1 (C). *, *P*<0.05; ***, *P*<0.001 compared with IgG-treated WT cells. No significant difference between IgG- and anti-MCP-1 Ab-treated MCP-1-KO cells (D). BMMs were transfected with sip47^phox^ or scRNA. Downregulation of p47^phox^ by siRNA was confirmed by RT-PCR and qPCR (E). The expression level obtained from scRNA-treated cells was set to be 1. After 24 h of transfection with siRNA, cells were stimulated with M-CSF for 2 d (mRNA) or 4 d (FACS) in order to determine CD11c (F) and for 2 d to measure ROS (G). *, *P*<0.05; **, *P*<0.01; ***, *P*<0.001 compared with scRNA-transfected WT cells. No significant difference was found in MCP-1-KO cells (F, G). Similar results were obtained in three independent experiments.

Since a lack of endogenous MCP-1 blunted M-CSF-induced CD11c in BMM, we attempted to investigate a mediator generated by MCP-1 to increase CD11c expression. Specific chemical inhibitors associated with M-CSF signaling pathways were evaluated for CD11c expression in the absence of MCP-1, compared with WT cells. As shown in **Figure 4A**, inhibition of PLC, Akt, and ERK decreased CD11c expression in WT cells, but no further decrease was observed in MCP-1-KO cells, suggesting that MCP-1 is required for activation of phospholipase C (PLC), transforming oncogene from Ak strain (Akt), and extracellular signal-regulated kinase (ERK) upon M-CSF stimulation. Consistent with this, phosphorylation of PLCγ2 reached a maximum level after a 3 d exposure to M-CSF in WT cells, whereas it was attenuated in MCP-1-deficient cells (**Figure 4B**). A similar pattern was found with Akt and ERK, as previously shown [23]. Decreased ROS levels by inhibition of nicotineamide adenine dinucleotide phosphate oxidase (NADPH oxidase) or exposure to an antioxidant, N-acetylcysteine (NAC) also decreased the expression level of CD11c in WT BMM, but no further decrease was observed in the absence of MCP-1 (**Figure 4A**). Exogenous H_2_O_2_ elevated CD11c in WT cells and recovered completely to the attenuated level of CD11c observed in MCP-1-KO cells (**Figure 4A**). These results suggested that ROS plays a positive role in elevating CD11c in M-CSF-stimulated BMM, and that MCP-1 is associated with ROS-induced CD11c expression. Next, we examined whether MCP-1 is associated with a long-lasting ROS level upon M-CSF stimulation. As shown in **Figure 4C**, M-CSF resulted in long-lasting ROS production in WT BMM, but the absence of MCP-1 reduced it. Blockage of MCP-1 decreased ROS upon M-CSF stimulation in WT cells, whereas no difference was observed in MCP-1-KO cells (**Figure 4D**), confirming that MCP-1 acts as a mediator to maintain ROS levels upon M-CSF stimulation. To confirm that MCP-1 mediates signaling to express CD11c via generation of ROS, siRNA to p47^phox^ (sip47^ phox^) which was a pool of 3 different siRNA duplexes or scrambled RNA (scRNA) was transfected to BMM. As shown in **Figure 4E**, treatment of sip47^ phox^ reduced the transcript of p47^ phox^. Down-regulation of p47^phox^ decreased the fraction of M-CSF-induced CD11c at both levels of transcript and surface protein (**Figure 4F**) and ROS levels (**Figure 4G**) in WT BMM, whereas no further decrease was observed in MCP-1-KO cells.

## Discussion

The underlying pathologies of metabolic syndrome, found in many postmenopausal women, are related to chronic inflammation, which is simultaneously associated with obesity [8]. OVX increased fat mass with elevated blood glucose [2], [3], [5] and IR [3], [5], [6], [7], although it is controversial. Vieira-Potter *et al.* [24] demonstrated that insulin sensitivity is more prominent even in the absence of increased adiposity at late stage of OVX. We have demonstrated that the absence of MCP-1 ameliorated chronic inflammation along with decreased fat mass induced by OVX. However, MCP-1 did not affect them in sham surgery mice, suggesting that MCP-1 may be a mediator of metabolic perturbation derived from loss of ovarian function.

OVX induced the infiltration of macrophages including CD11bF4/80 and CD11cF4/80 in visceral AT, whereas lack of MCP-1 reduced them. ATMs consist of at least two different phenotypes, including classically activated M1 macrophages and alternatively activated M2 macrophages, which are distinguished by the presence of CD11c, a surface M1 macrophage marker [11]. CD11c is a member of the β2 integrins and is expressed on a subpopulation of activated monocytes/macrophages [25]. CD11c-expressing cells generate pro-inflammatory cytokines, contributing to obesity-induced AT inflammation and IR [26]. CD11c deficiency ameliorates IR and improves glucose metabolism in obese mice [22], supporting the role of CD11c in IR. Thus, we have focused on CD11c expression to elucidate the role of MCP-1 in OVX-induced perturbation of glucose metabolism. We drew our attention to the chemotactic role of MCP-1. The lack of MCP-1 attenuated the influx of blood CD11c cells, which were significantly elevated by OVX, thus partly explaining the increase of CD11c cells in AT of OVX mice. Previously we have demonstrated that OVX increased serum MCP-1 [20]. OVX also resulted in increase of M-CSF compared with sham surgery, whereas the absence of MCP-1 reduced it. Accordingly, elevated levels of serum MCP-1 and M-CSF could augment CD11c in AT. A positive correlation between M-CSF and CD11c cells has been reported as following. CD11c cells are reduced in op/op mice, which lack functional M-CSF [27], confirming a requirement of M-CSF signaling in CD11c expression *in vivo*. Injection of M-CSF into mice augments CD11c cells in the blood [27], [28] and spleen [29]. Our *in vitro* data demonstrated that M-CSF induced CD11c expression in WT BMM, whereas a lack of MCP-1 attenuated it significantly. A blockade of MCP-1 in WT BMM decreased the ability of M-CSF to up-regulate CD11c expression, thus supporting the idea that MCP-1 mediates the up-regulation of CD11c by M-CSF. Taken together, our data suggest that an increased level of CD11c expression in AT upon OVX is associated with elevated levels of MCP-1 and M-CSF, which may create a permissive microenvironment for the cells to up-regulate CD11c expression as well as the increased recruitment of blood CD11c cells due to MCP-1.

Our data also show that OVX increased CD4 and CD8 T cells in AT, whereas the absence of MCP-1 reduced T cell accumulation, along with CD11c. Lack of CD11c decreased T cell accumulation and activation [30], suggesting a link between CD11c and T cells. T cells contribute to metabolic syndrome in HFD-induced obesity with increased accumulation and activation. Conversely granulocyte macrophage colony stimulating factor and interferon (IFN)-γ derived from activated T cells drive the generation of inflammatory CD11c cells [31], supporting the premise that infiltrated T cells also contribute to a microenvironment of highly inflammatory cells.

A previous clinical study shows that menopause causes oxidative stress [32]. Our study also showed that loss of ovarian function significantly increased serum ROS in mice. Elevated ROS are considered key contributing factors in the development of diabetes [33], [34] as well as in CD11c expression [35]. Our *in vitro* data demonstrated that H_2_O_2_ up-regulates the CD11c level of BMM upon M-CSF stimulation. Moreover, blockading ROS by NADPH oxidase inhibitor, diphenylene iodonium (DPI) or antioxidants, NAC reduced it, indicating that increased oxidative stress after OVX is highly associated with elevated CD11c in the AT of OVX mice. MCP-1 deficiency attenuated elevated serum ROS levels induced by OVX. Since no further decrease of CD11c by ROS blockading was found in the absence of MCP-1, the ability of MCP-1 to modulate ROS may be responsible for MCP-1-induced CD11c up-regulation. Exogenously-added MCP-1 further increased ROS levels induced by M-CSF. The association of MCP-1 with ROS production has been confirmed using siRNA. Knockdown of p47^phox^ using siRNA resulted in a decrease of CD11c expression as well as ROS level upon M-CSF stimulation, suggesting that ROS generation is responsible for CD11c induction. The absence of MCP-1 resulted in no further decrease of CD11c by sip47^phox^, confirming that MCP-1 contributes to the generation of the ROS required for up-regulation of CD11c. MCP-1 has been demonstrated to be responsible for the production of ROS in cardiomyocytes [36]. Moreover, it was found that the production of ROS and RNS induced by ischemia/reperfusion is attenuated in C-C chemokine receptor type 2 (CCR2)-KO mice [37], supporting the premise that MCP-1/CCR2 signals contribute to oxidative stress. M-CSF-stimulated CD11c expression in BMM was reduced by inhibition of PLCγ2, Akt, and ERK, whereas the absence of MCP-1 abolished this reduction, suggesting that the ability of MCP-1 to induce CD11c expression is via activation of PLCγ2, Akt, and ERK in BMM. Taken together, we showed that MCP-1 up-regulates CD11c expression in BMM via ROS generation and activation of PLCγ2, Akt, and ERK.

We have demonstrated a complex and critical role of MCP-1 in postmenopausal metabolic syndrome. OVX increased visceral fat and oxidative stress along with inflammation which was manifested by elevated M1 macrophages in AT. MCP-1-deficiency attenuated all of them *in vivo*. Besides CD11cF4/80, infiltration of immune cells including CD4 T and CD8 T cells in AT induced by OVX was significantly decreased by a lack of MCP-1. Up-regulation of CD11c-expressing cells in AT was caused by increased expression of CD11c cells in the tissue as well as by an elevated influx. The MCP-1-dependent CD11c subset may play an important role in maintaining inflammation, leading to disturbances of glucose metabolism. Our finding suggested that impaired ROS generation by MCP-1 deficiency reduced CD11c expression which led to inflammatory environment. However, we have not demonstrated how MCP-1 deficiency reduced fat mass which need to be investigated further. The ratios of fat mass and adipocyte volume of OVX mice from WT and MCP-1-KO mice were calculated to be 2.10 and 1.61, respectively, suggesting that reduced fat mass could be caused by decreased number as well as reduced volume of adipocytes. Impaired adipocyte differentiation affects fat mass [38]. It is possible that MCP-1 modulates fat mass via adipocyte differentiation, which has been increased by MCP-1-induced protein [39]. The important role of MCP-1 in IR and AT inflammation caused by OVX suggests that MCP-1 may be a novel therapeutic target for loss of ovarian function-induced metabolic syndrome in postmenopausal women.

## Materials and Methods

### Ethics Statement

All mice were handled in accordance with the guidelines of the Institutional Animal Care and Use Committee (IACUC) of the Immunomodulation Research Center (IRC), University of Ulsan. All animal procedures were approved by IACUC of IRC. The approval ID for this study is #2008–028.

### Animals and Study Design

MCP-1^−/−^ (MCP-1-KO) mice of a C57BL/6J genetic background were purchased from the Jackson Laboratory and were provided by the University of Ulsan, IRC. The offspring genotypes were determined by Southern blot analysis of DNA from tail biopsies. All mice were housed in the specific pathogen-free animal facility of the IRC. Six-week-old female MCP-1^+/+^ (WT) and MCP-1-KO mice were subjected to OVX (WT; 7, MCP-1-KO; 7) or sham operation (WT; 6, MCP-1-KO; 7). Food intake and body weight were monitored daily and weekly, respectively. At 12 weeks after OVX, mice were fasted for 6 h and sacrificed by cervical dislocation. Blood was taken by cardiac puncture, and tissues were immediately harvested. Blood glucose was measured with a commercially available enzyme assay kit (Asan Pharmacology, Hwa-Seong, Korea). Glucose and insulin tolerance tests were performed on 6 h-fasted mice. For glucose tolerance, animals were injected intraperitoneally with glucose (1 mg/kg), whereas for insulin tolerance, 0.75 munits/kg of recombinant human regular insulin (Eli Lilly, Indianapolis, IN) was injected intraperitoneally. Blood samples were drawn at 0, 15, 30, 60, and 120 min after glucose injection or 0, 15, 30, 60, and 90 min after insulin injection. Serum levels of MCP-1 and M-CSF were measured by a sandwich ELISA using coating anti-MCP-1 Ab or anti-M-CSF Ab and biotinylated anti-MCP-1 Ab or anti-M-CSF Ab, as recommended by the supplier (R & D Systems, Minneapolis, MN). Serum H_2_O_2_ was determined using an Amplex Red hydrogen peroxide/peroxidase assay kit (Invitrogen, Carlsbad, CA).

### SVC Isolation

Visceral fat pads were weighed, rinsed three times in phosphate-buffered saline (PBS), and minced with FACS buffer (PBS with 1% BSA). Tissue suspensions were centrifuged at 500×g for 5 min, and then treated with type 2 collagenase (1 mg/ml, Sigma Chemical. St Louis, MO) for 90 min at 37°C with shaking. Cell suspensions were filtered through a 100 µm filter and centrifuged at 500×g for 5 min. SVC pellets were incubated with RBC lysis buffer (eBioscience, San Diego, CA) for 5 min, centrifuged at 300×g for 5 min, and resuspended in FACS buffer. SVC was incubated with Fc blocker for 20 min at 4°C before staining fluorescently-labeled Abs against CD11b, CD11c, F4/80, CD4, and CD8 (eBioscience). Cells were gently washed twice, resuspended in FACS buffer, and analyzed using a FACSCanto II flow cytometer (BD Sciences, San Jose, CA).

### Cell Preparation

Femora and tibiae were removed aseptically and dissected free of adherent soft tissue. The bone ends were cut, and the marrow cavity was flushed with α-MEM from one end of the bone, using a sterile 21-gauge needle. The bone marrow was further agitated using a Pasteur pipette to obtain a single-cell suspension, which was washed twice and incubated on plates with M-CSF (20 ng/ml) (R&D Systems) for 16 hours. Non-adherent cells were then harvested, layered on a Ficoll-Hypaque gradient, and cultured for two more days, by which time large populations of adherent monocyte/macrophage-like cells had formed on the bottoms of the culture plates, as previously described [23]. The few non-adherent cells were removed by washing the dishes with PBS, and the adherent cells (BMM) were harvested and seeded on plates. The adherent cells were analyzed to be negative for CD3 and CD45R and positive for CD11b and F4/80. The absence of contaminating stromal cells was confirmed by lack of cell growth in the absence of M-CSF. Additional medium with M-CSF was added and later refreshed on day 3. After incubation for the indicated times, the cells were analyzed using a FACSCantor. BMMs were transfected with small interfering RNA (siRNA) against p47^phox^ (sip47^phox^) or scrambled siRNA (scRNA) (Santa Cruz Biotech., Santa Cruz, CA), using Lipofectamine™ RNAiMAX (Invitrogen), and further analyzed.

### Quantitative PCR

Total RNA from BMMs incubated with M-CSF for the indicated time period was extracted using Trisol solution (GIBCO, Life Technol.) and reverse-transcribed with oligo-dT and Superscript I enzyme (Invitrogen). Quantitative PCR was carried out using SYBR Green 1 Taq polymerase (Qiagen, Hilden, Germany) and appropriate primers on a DNA Engine Opticon Continuous Fluorescence Detection System (MJ Research Inc.). The specificity of each primer pair was confirmed by melting curve analysis and agarose-gel electrophoresis. Relative copy numbers compared to RPS3 were calculated using 2^−ΔΔCt^. The primer sequences (forward and reverse, respectively) used were as follows: 5′-ctggatagcctttcttctgctg-3′ and 5′-gcacactgtgtccgaactc-3′ (CD11c); 5′-gggtcattcagaggcacactg-3′ and 5′-ctggctgtcctgtcaaggct-3′ (CD163); 5′-ctccaagccaaagtccttaga-3′ and 5′-aggagctgtcattagggacatc-3′ (arginase-1); 5′-atcagagagttgaccgcagttg-3′ and 5′-aatgaaccgaagcacaccatag-3′ (RPS3).

### Intracellular ROS Detection

The intracellular formation of ROS was detected using the fluorescence probe, 2′,7′-dichlorofluorescein diacetate (H2DCFDA) (Molecular Probe). After the BMM were cultured under the different experimental conditions for 2 d, the cells were washed, trypsinized, suspended in PBS, loaded with H2DCFDA, and incubated at 37°C for 30 min. The measurement of intracellular ROS was performed using a flow cytometer with a FACSCantor.

### Western Blotting

Western blots were probed with Abs against the phosphorylated and total forms of PLCγ2 (Cell Signaling, Danvers, MA) and then incubated with the appropriate peroxidase-conjugated secondary Ab (Santa Cruz Biotech).

### Statistical Analysis

Values are expressed as mean ± SEM. Student’s *t*-test was used to evaluate differences between samples of interest and corresponding controls. Differences between groups were assessed by one-way or two-way ANOVA, followed by Bonferroni post-tests. A *P*-value less than 0.05 was considered statistically significant.
